# Dipeptidyl Peptidase 3 Activity as a Promising Biomarker of Bone Fragility in Postmenopausal Women

**DOI:** 10.3390/molecules27123929

**Published:** 2022-06-19

**Authors:** Ciro Menale, Gaia Tabacco, Anda Mihaela Naciu, Maria Lucia Schiavone, Francesca Cannata, Emanuela Morenghi, Cristina Sobacchi, Andrea Palermo

**Affiliations:** 1 IRCCS Humanitas Research Hospital, Via Manzoni 56, 20089 Rozzano, Italy; ciro.menale@unina.it (C.M.); marialucia.schiavone@humanitasresearch.it (M.L.S.); 2 Department of Clinical Medicine and Surgery, University of Naples “Federico II”, Via Pansini 5, 80131 Napoli, Italy; 3 Unit of Metabolic Bone and Thyroid Disorders, Fondazione Policlinico Universitario Campus Bio-Medico, 00128 Rome, Italy; g.tabacco@unicampus.it (G.T.); a.naciu@unicampus.it (A.M.N.); a.palermo@unicampus.it (A.P.); 4 Unit of Endocrinology and Diabetes, Campus Bio-Medico University of Rome, 00128 Rome, Italy; f.cannata@unicampus.it; 5 Biostatistics Unit, IRCCS Humanitas Research Hospital, Via Manzoni 56, 20089 Rozzano, Italy; emanuela.morenghi@humanitas.it; 6 CNR-IRGB, Milan Unit, Via Fantoli 16/15, 20138 Milan, Italy

**Keywords:** *Dpp3*, oxidative stress, bone mineral density, osteoporosis, fragility fracture, serum biomarker

## Abstract

The dipeptidyl peptidase 3 (*Dpp3*) is a ubiquitous zinc-dependent aminopeptidase, participating in the activation or degradation of signaling peptides and in the Keap1–Nrf2 antioxidant pathway. The absence of *Dpp3* in the *Dpp3* knockout mouse model causes increased osteoclast activity, altered osteogenic function, sustained oxidative stress in the bone tissue, and bone loss. We aimed to assess the association of *Dpp3* activity with bone fragility in postmenopausal osteoporosis and the impact of denosumab on enzymatic activity. We conducted a two-phase study including 69 postmenopausal women with severe osteoporosis and 36 postmenopausal women without osteometabolic conditions, as controls (cross-sectional phase). Subjects with severe osteoporosis were assessed at baseline and 14 days after the first denosumab administration (prospective phase). The results showed significant reduction in serum *Dpp3* activity (expressed as nmoles of formed product/mg proteins/min) in patients vs. controls (0.791 ± 0.232 vs. 1.195 ± 0.338; *p* < 0.001), and significant association with bone mass at the femoral neck (r = 0.28, *p* = 0.02) in patients prior to treatment. We found a negative correlation between C-terminal telopeptide (CTX) or N-terminal pro-peptide of type 1 procollagen (P1NP) levels and *Dpp3* activity (respectively, r = −0.29, *p* = 0.012; and r = −0.2572, *p* = 0.033). *Dpp3* activity did not change after denosumab injection. Our findings support a critical role played by *Dpp3* in bone homeostasis as a potential bone protective factor. Additional clinical studies in larger cohorts might explore the implementation of *Dpp3* assessment as a biomarker of bone health status.

## 1. Introduction

The dipeptidyl peptidase 3 (*Dpp3*) is a ubiquitous zinc-dependent aminopeptidase, highly expressed in human cells and conserved among higher vertebrates [1]. It cleaves dipeptides from the N-terminus of oligopeptides, thereby participating in the activation or degradation of signaling peptides, such as angiotensin 2 and enkephalins, involved, respectively, in the renin–angiotensin system and blood pressure regulation, and in nociception [2]. Moreover, *Dpp3* is a regulator of the cellular oxidative stress response through the Keap1–Nrf2 antioxidant pathway; in this context, binding of *Dpp3* to Keap1 releases the transcription factor Nrf2, which thus escapes degradation by the 26S proteasome and migrates into the nucleus, where it drives the expression of various antioxidant enzymes [3]. *Dpp3* antioxidant activity has been particularly associated with inflammation and carcinogenesis [4,5,6], and recently with bone metabolism in a mouse model [7]. In fact, Menale et al. demonstrated that adult mice lacking *Dpp3* (*Dpp3* knockout (KO) mice) presented bone mass loss owing to increased osteoclast activity, as well as altered osteogenic function secondary to sustained oxidative stress in the bone tissue, and that, in an estrogen deprivation model, *Dpp3* KO female mice had a greater decrease in bone mass, compared with wild-type animals. This pointed to a non-redundant role of *Dpp3* in the maintenance of bone homeostasis.

Fragility fractures are prevalent in postmenopausal women resulting in bone-associated morbidities, reduced quality of life, increased mortality, and healthcare costs. Accordingly, owing to the estimated number of people affected by osteoporosis (about 200 million worldwide) and the considerable social and healthcare costs associated, this disease clearly constitutes a global (though often overlooked) public health problem [8].

Bone mineral density (BMD) evaluated via dual-energy X-ray absorptiometry (DXA) is the most common parameter to identify patients at risk of fragility fractures. A T-score (i.e., standard deviation difference from mean values of sex-matched young, healthy individuals) equal to or below −2.5 standard deviation (SD) is considered diagnostic for osteoporosis; however, most individuals that sustain fragility fractures are above this cutoff, which highlights an incongruity with serious drawbacks. To overcome this issue, other imaging techniques have been developed, but they are mostly employed as research tools [9].

Considering this scenario, the identification of an early biomarker of bone impairment to be easily assessed in the routine clinical practice is expected to improve patients’ management, prevent fractures, preserve the quality of life, and alleviate healthcare costs.

*Dpp3* is primarily a cytosolic protein; however, it is also detectable extracellularly in several biological fluids, most importantly in human plasma and serum in pathophysiological conditions [10,11,12]. In the framework of bone pathology, there are no clinical studies aimed at investigating circulating *Dpp3* activity as a marker of bone loss in postmenopausal women with fragility fractures. Hence, in our research, we investigated whether *Dpp3* had a role in skeletal fragility in humans. To answer this question, we measured serum *Dpp3* activity in a cohort of postmenopausal women with severe osteoporosis. Furthermore, we evaluated if a powerful antiresorptive agent can affect *Dpp3* activity.

## 2. Results

### 2.1. Cross-Sectional Evaluation

The characteristics of the two cohorts of individuals are shown in Table 1. Study groups were homogeneous for age at menopause, calcium, phosphate, 25-OH vitamin D, and parathyroid hormone (PTH) levels. On the other hand, subjects affected by osteoporosis were older than controls (71.93 ± 8.21 years vs. 66.64 ± 8.73 years, *p* < 0.003) and had lower glomerular filtration rate (GFR, 77.78 ± 21.42 vs. 88.51 ± 18.82 mL/min/m^2^, *p* < 0.003), though not clinically suggestive of kidney disorders. BMD (g/cm^2^) was statistically different between the two groups at each site assessed (lumbar spine, L1–L4: 0.800 ± 0.158 vs. 0.939 ± 0.131, *p* = 0.001; femoral neck, FN: 0.624 ± 0.108 vs. 0.718 ± 0.112, *p* = 0.001; total hip, TH: 0.697 ± 0.126 vs. 0.874 ± 0.078, *p* = 0.001; all the comparisons are indicated as values in patients compared with controls). Serum carboxy-terminal collagen cross-links (CTX) and N-terminal pro-peptide of type 1 collagen (P1NP) were measured only in the patient cohort (0.371 ± 0.18 and 50.51 ± 24.57 pg/mL, respectively, at the patient’s enrollment).

**Figure 1:**
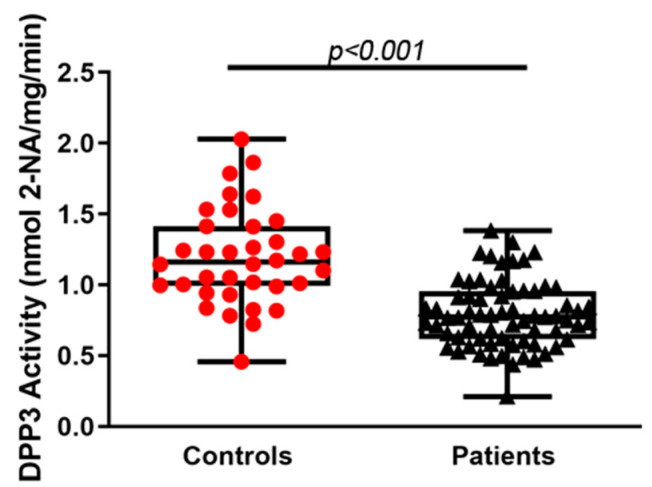
*Dpp3* activity in the serum of controls (*n* = 36) and postmenopausal women with severe osteoporosis (*n* = 69) at baseline. Statistical significance was calculated using two-tailed Mann–Whitney test; *p* < 0.001.

Serum *Dpp3* activity, indicated as nmol of β-naphthylamine (2-NA) released by hydrolysis of the substrate Arg-Arg-β-naphthylamide/mg proteins/min, was significantly lower in patients, compared with controls (0.791 ± 0.232 vs. 1.195 ± 0.338; *p* < 0.001) (Figure 1), with a regression coefficient of 0.40 (95% CI 0.29–0.51). This association remained significant after correction for age (regression coefficient 0.36, 95% CI 0.24–0.47, *p* < 0.001).

**Figure 2:**
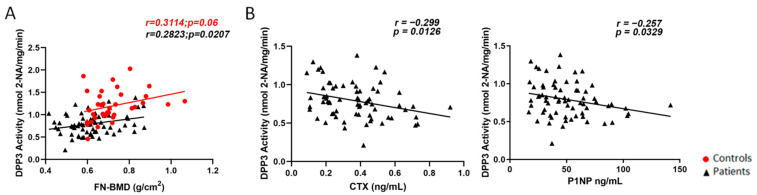
(**A**) Pearson correlation analysis (two-tail) between femoral neck BMD (FN-BMD) and circulating *Dpp3* activity in our cohorts of controls (depicted as red circles, *n* = 36) and patients (depicted as black triangles, *n* = 67); (**B**) Pearson correlation analysis (two-tail) between the bone turnover markers CTX (**left**) and P1NP (**right**) and circulating *Dpp3* activity in our cohort of osteoporotic women at baseline (*n* = 69).

Regarding densitometric parameters, we found a significant positive correlation between FN-BMD and *Dpp3* activity in patients (r = 0.28, *p* = 0.02) (Figure 2A); as regards TH-BMD, a negative correlation with *Dpp3* activity was observed in patients (r = −0.32, *p* = 0.014), at variance with controls, where we found a positive correlation (r = 0.38, *p* = 0.019) (Supplementary Figure S1A). No significant correlation was found between L1–L4 BMD and *Dpp3* activity (Supplementary Figure S1B).

With respect to bone turnover markers, we found a negative correlation between CTX or P1NP levels and *Dpp3* activity (respectively, r = −0.29, *p* = 0.012; and r = −0.2572, *p* = 0.033; Figure 2B).

**Figure 3:**
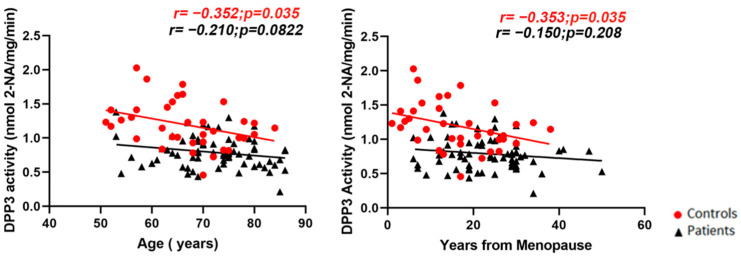
Pearson correlation analysis (two-tail) between age (**left** panel) or years from menopause (**right** panel) and *Dpp3* activity in the serum of controls (depicted as red circles, *n* = 36) and postmenopausal women with severe osteoporosis (depicted as black triangles, *n* = 69) at baseline.

We observed a significant negative correlation between both age and years from menopause and *Dpp3* activity in controls (respectively, r = −0.3521, *p* = 0.0352; and r = −0.3532, *p* = 0.0346) but not in the patient population (Figure 3).

Finally, we found no significant correlation between vitamin D, PTH, calcium, and phosphate levels and *Dpp3* activity in the sera of osteoporotic patients. Furthermore, no significant association was revealed with the number of fractures in postmenopausal women (Supplementary Table S1).

### 2.2. Prospective Evaluation

**Figure 4:**
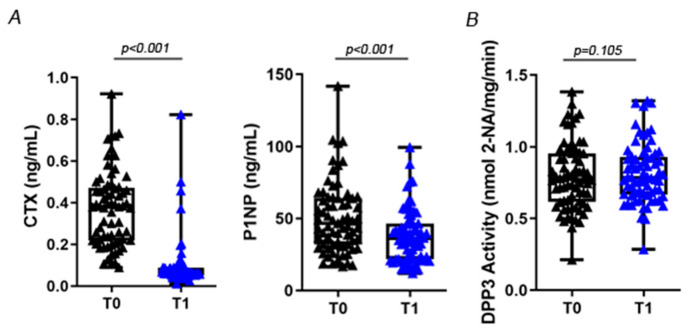
Serum CTX and P1NP levels (**A**) and *Dpp3* activity (**B**) in our cohort of postmenopausal women with severe osteoporosis at baseline (T0, black triangles) and 2 weeks after the first dose of the antiresorptive drug denosumab (T1, blue triangles).

As expected, we found a significant reduction in CTX (0.370 ± 0.180 ng/mL vs. 0.097 ± 0.122 ng/mL, *p* < 0.001) and P1NP (50.51 ± 24.57 ng/mL vs. 37.80 ± 18.21 ng/mL; *p* < 0.001) 14 days after injection of the monoclonal antibody against receptor activator of NF-kB ligand (RANKL) denosumab (Figure 4A). On the other hand, *Dpp3* activity did not change after denosumab treatment (0.791 ± 0.232 vs. 0.815 ± 0.207; *p* = 0.105) (Figure 4B).

## 3. Discussion

Despite the attention gained by *Dpp3* since its discovery at the end of the 1960s [13], several aspects related to its function remain elusive [14]. For example, a poorly investigated topic is *Dpp3* function in the context of bone biology, which has been addressed only recently by Menale et al., who showed that the absence of *Dpp3* in the mouse model *Dpp3* KO caused bone loss owing to increased osteoclast activity and impaired bone quality, particularly in female mice after ovariectomy [7]. For the human counterpart, the present report is the first study that evaluated *Dpp3* activity in a cohort of subjects with severe osteoporotic bone fragility. In this context, we found significantly lower *Dpp3* levels in patients, compared with a control population with no osteometabolic disorders, in agreement with previous evidence in mice.

Moreover, we found a positive correlation between FN-BMD and *Dpp3* activity in severe osteoporosis; this is relevant since it has been well demonstrated that FN-BMD is the most accurate DXA parameter to estimate the fracture risk [15]. The finding of an inverse correlation between TH-BMD and *Dpp3* activity in the patients compared with controls is puzzling, and at present, we cannot provide an explanation. Expanding the study to a larger population might correct or confirm this evidence.

The negative correlation between standard bone turnover markers and *Dpp3* enzymatic activity in osteoporotic women prior to antiosteoporotic treatment strengthened *Dpp3* association with bone metabolism. A single administration of a potent antiresorptive agent (denosumab) caused a significant reduction of serum CTX and P1NP levels two weeks after treatment, as expected, while did not significantly affect *Dpp3* activity in patients. We cannot exclude changes in *Dpp3* levels at later time points after drug administration or after prolonged treatment. It would be also interesting to assess changes in *Dpp3* levels after treatment with different antiresorptive as well as osteoanabolic drugs [16].

Overall, our data indicate that *Dpp3* preserves bone health, which sounds reasonable based on evidence of reduced Nrf2 and HO-1 expression and increased lipid peroxidation in the bone tissue lacking *Dpp3* and on the well-known detrimental effect of sustained inflammation and oxidative stress on bone homeostasis [17].

The negative correlation between age and years from menopause, and *Dpp3* activity in the control population suggests that *Dpp3* activity progressively decreases with age in healthy individuals and approaches the (already low) levels measured in the patients. In addition, the absence of correlation with age in the patients, compared with the controls, suggested that osteoporosis owing to estrogen withdrawal had a greater effect on *Dpp3* activity, compared with the impact of aging. Indeed, as mentioned above, in the *Dpp3* KO mouse model, estrogen withdrawal aggravated bone loss, compared with the wild-type counterpart. Accordingly, an important upregulation of *Dpp3* expression by estrogens has been demonstrated in the liver of wild-type mice treated with 17β-estradiol [18].

Notably, we revealed here that lower circulating *Dpp3* enzymatic levels are associated with a worse skeletal phenotype; conversely, in sepsis, cardiogenic shock, heart failure, and acute kidney injury the correlation goes in the opposite direction, with higher levels resulting in more severe disease and reduced survival rate [19,20,21,22,23,24,25]. In these conditions, angiotensin 2, the primary effector of the renin–angiotensin system (RAS), is recognized as the key substrate of *Dpp3* activity and clinical evidence has been interpreted based on the modulation of the RAS by *Dpp3*, and the resulting impact on hemodynamics and on the physiology of the cardiovascular system. By contrast, the specific substrate(s) of *Dpp3* enzymatic activity in the bone is not known yet, and the skeletal phenotype in the *Dpp3* KO mice has been essentially related to sustained oxidative stress. In fact, the Keap1–Nrf2 antioxidant system, which comprises also *Dpp3* among its activators, is known to exert both protective and detrimental effects, in cancer [4] as well as in bone biology, acting both on the osteoclast and on the osteoblast lineage [26].

All these data suggest that *Dpp3* participates in a complex and pleiotropic network that requires to be better elucidated. Dissection of basic *Dpp3*-related pathophysiological mechanisms will likely have implications for the diagnosis and treatment of human diseases.

Our study has some limitations—namely, the cross-sectional design, the limited number of controls, and the short follow-up period for the prospective evaluation; on the other hand, it constitutes the starting point for investigation in a larger cohort of osteoporotic patients. In parallel, it would be interesting to test the hypothesis of a bone-protective role of *Dpp3* in other pathological conditions resulting in bone deterioration. As a perspective, serum *Dpp3* activity measurement might be easily implemented as a biomarker of bone fragility in routine clinical practice.

## 4. Materials and Methods

### 4.1. Study Population and Design

We performed a two-phase study with a cross-sectional and a prospective evaluation, overall including 105 Caucasian subjects. The cross-sectional phase was conducted on 69 postmenopausal women affected by severe osteoporosis and 36 postmenopausal women with either osteopenia [27] or normal BMD, as controls. The prospective phase of the study included 69 subjects with severe osteoporosis. In detail, after baseline evaluation, all subjects started denosumab (Prolia^®^, Amgen Europe; 60 mg subcutaneously, every six months) as a treatment for severe osteoporosis. Fourteen days after the first denosumab administration, subjects underwent blood drawing for evaluation of bone turnover markers and *Dpp3* activity.

Participants were consecutively enrolled at Fondazione Policlinico Campus Bio-Medico of Rome. Inclusion criteria for the cases were postmenopausal status, lumbar spine and/or non-dominant total hip and/or femoral neck T-score <2.5, at least one vertebral fragility fracture, and absence of ongoing or previous antiosteoporotic treatments. Inclusion criteria for controls were postmenopausal status, normal or osteopenic BMD at L1–L4 and non-dominant TH and FN, and absence of osteoporotic fractures.

Exclusion criteria for all participants were the presence of any condition that can affect bone and calcium metabolism—namely, early menopause, chronic kidney disease (glomerular filtration rate, GFR, <30 mL/min/1.73 m^2^), a history of possible high-energy vertebral fractures, metabolic bone diseases, sarcoidosis, inflammatory bowel diseases, rheumatic diseases, musculoskeletal disorders other than osteoporosis and medications such as bisphosphonates, teriparatide, estrogens, corticosteroids, aromatase inhibitors, or other drugs that could interfere with bone metabolism.

### 4.2. Biochemical Analysis

In the morning, fasting blood samples were collected for the evaluation of serum total calcium, phosphate, creatinine, and 25 OH vitamin D, using automated methods. Intact PTH was measured via an immunochemiluminometric assay using a Modular E170 automatic analyzer (Roche Diagnostics, Indianapolis, IN, USA). Serum levels of β-cross-laps (CTX) were assayed by the Cobas β-CrossLaps (ECLIA; β-CrossLaps/Serum, Roche Diagnostics, Basel, Switzerland), which uses two monoclonal antibodies against β-cross-linked CTX, according to the manufacturer’s protocol. Serum levels of P1NP were analyzed with a Cobas Total P1NP (ECLIA; Roche Diagnostics) automated analyzer.

### 4.3. Dpp3 Activity Measurement

*Dpp3* enzymatic activity in the sera of patients and controls was measured as previously reported [7], with minor modifications. Briefly, serum protein concentration was determined using the DC Protein Assay kit (Bio-Rad, Hercules, CA, USA), according to the manufacturer’s instructions. Then, a volume of serum corresponding to about 50 μg of total proteins was assayed with 0.04 mM Arg-Arg-β-naphthylamide (Sigma-Aldrich, St. Louis, MO, USA) in Tris-HCl, pH 8.5, at 37 °C. The reaction was stopped by adding 2 M acetate buffer, pH 4.5, containing 10% Tween and 1.5 mg/mL Fast Blue B Salt (all chemicals from Sigma-Aldrich). The absorbance of the released product (β-naphthylamine) was measured at 530 nm, using a Synergy^TM^ H4 Microplate Reader. The enzymatic activity was expressed as nmol of β-naphthylamine (2-NA)/mg proteins/min).

### 4.4. Dual-Energy X-ray Absorptiometry (DXA)

BMD was measured by DXA at L1–L4, TH, and FN (Hologic Discovery QDR Instrument, Marlborough, MA, USA). We reported data for absolute BMD. All scans were performed according to the International Society for Clinical Densitometry (ISCD) guidelines [28]. Fractured vertebrae and vertebrae with structural changes (T-score difference with the adjacent vertebra >1.0) were excluded from the analysis.

### 4.5. Vertebral Fracture Assessment

Vertebral Fractures (VFs) were assessed on conventional spinal radiographs (T4-L4) in the lateral and the anteroposterior projections and using the Genant semiquantitative method (grade 1, mild; grade 2, moderate; grade 3, severe) [29].

### 4.6. Ethics

The study was conducted in compliance with the Declaration of Helsinki and the International Conference on Harmonization Principles of Good Clinical Practice. The local ethics committee approved the research protocol, and all participants gave informed consent prior to inclusion in the study.

### 4.7. Statistical Analysis

Data were described as mean and standard deviation. Data distribution adherence to Gaussian distribution was evaluated using the Kolmogorov–Smirnov test. The difference between case and control groups was explored with an unpaired Student’s *t*-test if the distribution was approximately Gaussian, or with Mann–Whitney test otherwise. For Student’s *t*-test, variance equality was determined according to Levene’s test. Correlations between *Dpp3* activity and CTX, P1NP, calcium phosphate, PTH, 25 OH-Vitamin D, FN-, TH-, and L1–L4 BMD were tested using Pearson’s correlation. The association of *Dpp3* activity and case and control was explored with linear regression, singularly considered and age corrected. Results were then expressed as a regression coefficient with a 95% confidence interval (95% CI). A *p* value lower than 0.05 was considered significant.

## 5. Conclusions

The results of the present clinical study, together with previous evidence in a relevant mouse model, point to a critical role played by *Dpp3* in bone homeostasis as a bone protective factor. Additional clinical studies in larger cohorts of osteoporotic women, as well as in patients affected by different bone pathologies, might extend further the range of diseases in which *Dpp3* is involved and its possible exploitation and implementation as a biomarker.

## Figures and Tables

**Table 1:** Baseline demographic, clinical, and laboratory characteristics of the study population.

	Patients (*n* = 69)	Controls (*n* = 36)	*p* Value
Age, years	71.93 ± 8.21	66.64 ± 8.73	0.003
Age at menopause, years	49.01 ± 5.16	50.22 ± 2.95	0.131
Calcium, mg/dL (8.4–10.2)	9.36 ± 0.43	9.45 ± 0.37	0.256
Phosphate, mg/dL (2.3–4.7)	3.63 ± 0.47	3.81 ± 0.41	0.057
25-OH Vitamin D, ng/mL	30.74 ± 13.25	29.91 ± 15.14	0.773
PTH, pg/mL (13–85)	52.81 ± 24.42	49.06 ± 15.32	0.338
GFR, mL/min/m^2^	77.78 ± 21.42	88.51 ± 18.82	0.013
CTX, ng/mL	0.371 ± 0.18	n.a.	n.a.
P1NP, ng/mL	50.51 ± 24.57	n.a.	n.a.
L1–L4 BMD, g/cm^2^	0.800 ± 0.158	0.939 ± 0.131	0.001
FN-BMD, g/cm^2^	0.624 ± 0.108	0.718 ± 0.112	0.001
TH-BMD, g/cm^2^	0.697 ± 0.126	0.874 ± 0.078	0.001

Normal ranges for the indicated parameters are reported in brackets. Data for patients and controls are indicated as mean ± SD. The difference between the means was analyzed using the unpaired Student’s *t*-test. Abbreviations: PTH, parathyroid hormone; GFR, glomerular filtration rate; CTX, beta cross-laps (C-terminal cross-linking telopeptide); P1NP, pro-collagen type 1 N-terminal pro-peptide; BMD, bone mineral density; FN, femoral neck; TH, total hip; n.a., not assessed.

## Data Availability

All data supporting the findings of this study are present in the main text and/or in the Supplementary Information.

## Supplementary Materials

The following supporting information can be downloaded at: https://www.mdpi.com/article/10.3390/molecules27123929/s1, Figure S1: Pearson correlation analysis between BMD at hip or spine and circulating *Dpp3* activity, Table S1: Correlation analysis between cDPP3 enzymatic activity in our cohort of postmenopausal women with severe osteoporosis and biochemical clinical parameters that did not reach statistical significance after linear regression and Pearson’s correlation.

## Sample Availability

Samples of the compounds are available from the authors.

## Abbreviations

*Dpp3*	Dipeptidyl peptidase 3
Keap1	Kelch-like ECH associated protein-1
Nrf2	Nuclear factor erythroid 2-related factor-2
CTX	C-terminal telopeptide
P1NP	N-terminal pro-peptide of type 1 procollagen
BMD	Bone mineral density
DXA	Dual-energy X-ray absorptiometry
SD	Standard deviation
PTH	Parathyroid hormone
GFR	Glomerular filtration rate
FN	Femoral neck
TH	Total hip
2-NA	β-Naphthylamine
RANKL	Receptor activator of NFkB ligand
HO-1	Heme oxygenase 1
RAS	Renin–angiotensin system
