# Supplementary material for: Dipeptidyl Peptidase 3 Activity as a Promising Biomarker of Bone Fragility in Postmenopausal Women
Source: Molecules. 2022 Jun 19;27(12):3929. doi: 10.3390/molecules27123929 (PMC9227407; doi:10.3390/molecules27123929)
Supplement: Supplementary file 1 [file molecules-27-03929-s001.zip › molecules-1761206-supplementary.pdf]

*Article*

# Dipeptidyl Peptidase 3 Activity as a Promising Biomarker of Bone Fragility in Postmenopausal Women

**Ciro Menale [1,2,*], Gaia Tabacco [3,4,*], Anda Mihaela Naciu [3,4], Maria Lucia Schiavone [1], Francesca Cannata [4], Emanuela Morenghi [5], Cristina Sobacchi [1,6,*], Andrea Palermo [3,4]**

## Supplementary Information

### Supplementary Table S1

| Clinical parameters | Pearson r | R squared | P value (two-tailed) |
|---|---|---|---|
| Vitamin D | -0.036 | 0.0013 | 0.77 |
| PTH | 0.101 | 0.0102 | 0.41 |
| Calcium | -0.128 | 0.0164 | 0.30 |
| Phosphorus | 0.010 | 0.0001 | 0.93 |
| Number of fractures | -0.047 | 0.0022 | 0.70 |

**Supplementary Table S1**. Correlation analysis between cDPP3 enzymatic activity in our cohort of postmenopausal women with severe osteoporosis and biochemical clinical parameters that did not reach statistical significance after linear regression and Pearson's correlation.

Figure 1 supplementary

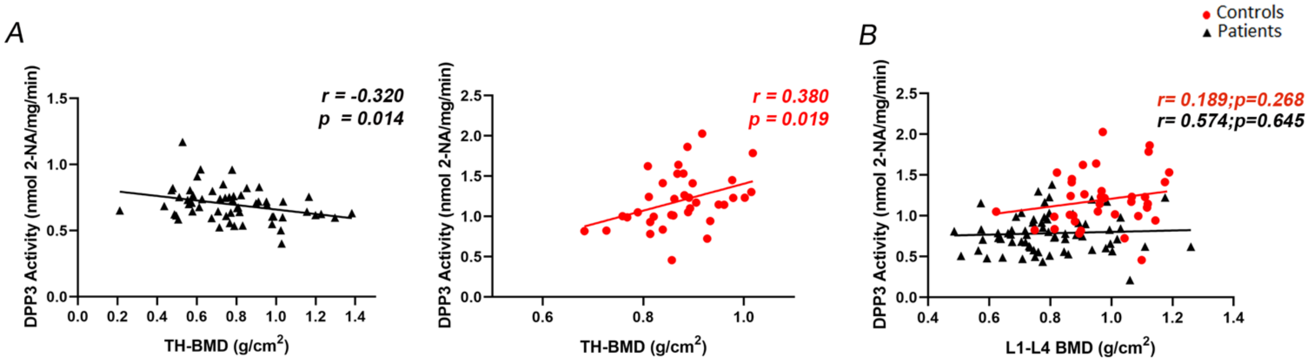


**Supplementary Figure S1**. Pearson correlation analysis between BMD at hip or spine and circulating DPP3 activity. (A) Pearson correlation analysis (two-tail) between Total Hip BMD (TH-BMD) and circulating DPP3 activity in our cohorts of patients (depicted as black triangles, n=67) and controls (depicted as red circles, n=36). (B) Pearson correlation analysis (two-tail) between spine BMD (L1-L4 BMD) and circulating DPP3 activity in our cohorts of controls (depicted as red circles, n=36) and patients (depicted as black triangles, n=67).
